# Supplementary material for: Trans-Encapsidation of Foot-and-Mouth Disease Virus Genomes Facilitates Escape from Neutralizing Antibodies
Source: Viruses. 2022 May 27;14(6):1161. doi: 10.3390/v14061161 (PMC9229618; doi:10.3390/v14061161)
Supplement: Supplementary file 1 [file viruses-14-01161-s001.zip › viruses-1680640-supplementary.pptx]

## Slide 1
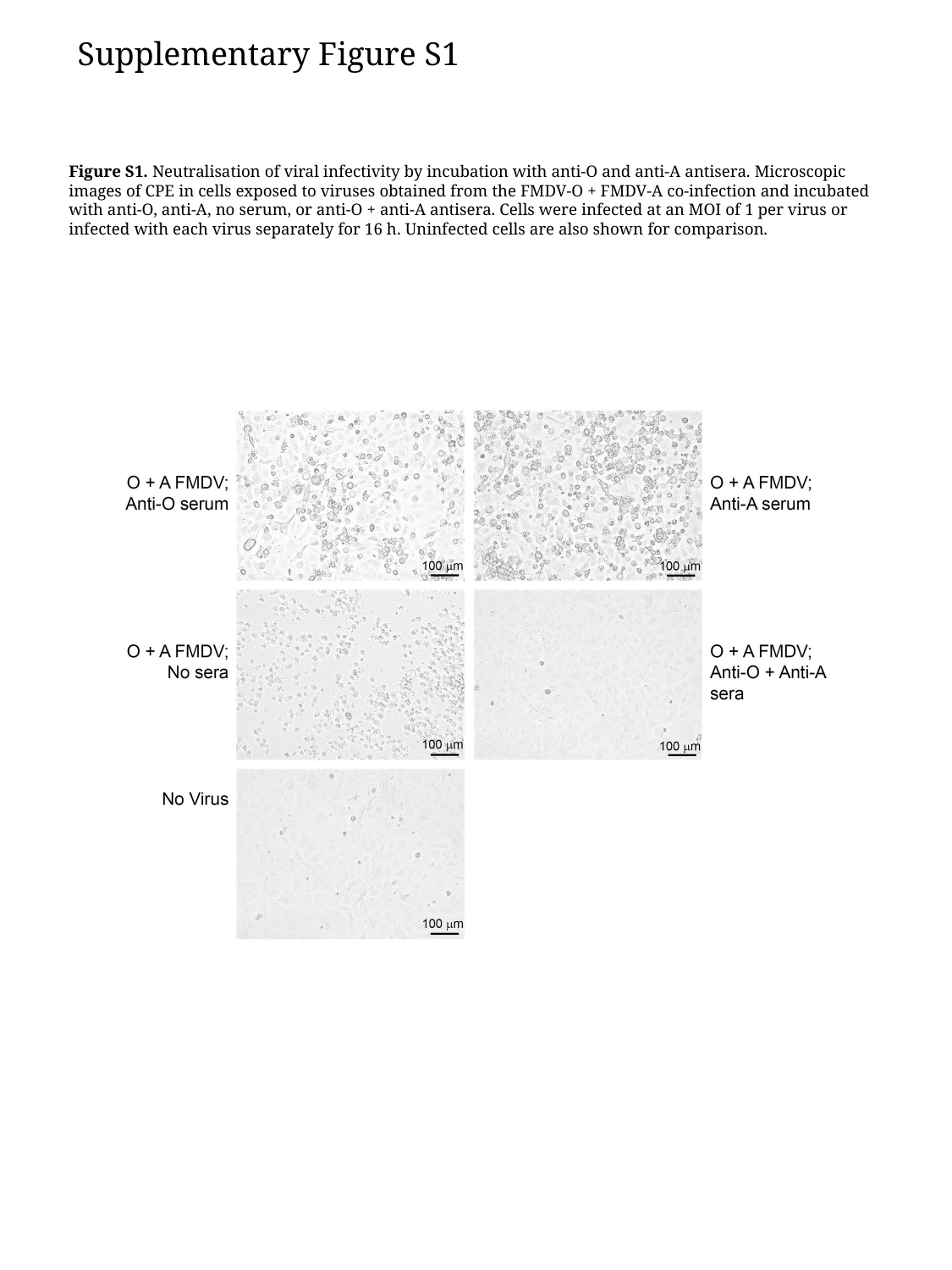

Supplementary Figure S1
Figure S1. Neutralisation of viral infectivity by incubation with anti-O and anti-A antisera. Microscopic images of CPE in cells exposed to viruses obtained from the FMDV-O + FMDV-A co-infection and incubated with anti-O, anti-A, no serum, or anti-O + anti-A antisera. Cells were infected at an MOI of 1 per virus or infected with each virus separately for 16 h. Uninfected cells are also shown for comparison.

## Slide 2
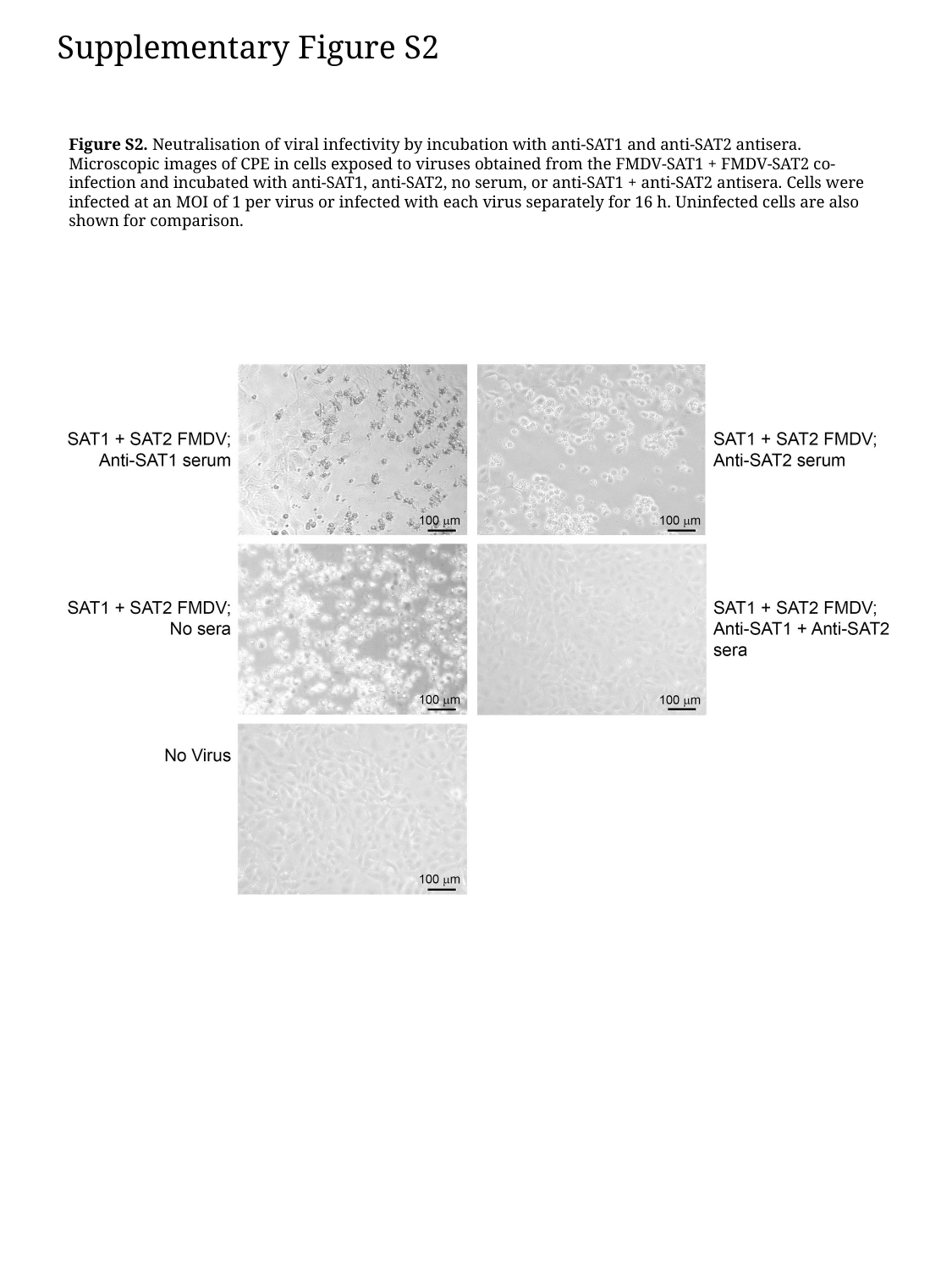

Supplementary Figure S2
Figure S2. Neutralisation of viral infectivity by incubation with anti-SAT1 and anti-SAT2 antisera. Microscopic images of CPE in cells exposed to viruses obtained from the FMDV-SAT1 + FMDV-SAT2 co-infection and incubated with anti-SAT1, anti-SAT2, no serum, or anti-SAT1 + anti-SAT2 antisera. Cells were infected at an MOI of 1 per virus or infected with each virus separately for 16 h. Uninfected cells are also shown for comparison.
